# The bone cement with screw augmentation surgical technique in a 3D-printed customized total knee arthroplasty for a patient with severe bone defect

**DOI:** 10.1093/jscr/rjaf884

**Published:** 2025-11-05

**Authors:** Argyris Hadjimichael, Petros Leptos, Jim D Georgoulis, Angelos Kaspiris

**Affiliations:** Department of Orthopedic Surgery, Saint Mary's and John's Polyclinic, PO 2045, Karditsis 2, Strovolos, Nicosia, Cyprus; Department of Orthopedic Surgery, Saint Mary's and John's Polyclinic, PO 2045, Karditsis 2, Strovolos, Nicosia, Cyprus; First Department of Orthopedic Surgery, National and Kapodistrian University of Athens, PO 12462, Rimini 1, Haidari, Athens, Greece; Department of Orthopedic Surgery, KAT Attica General Hospital, National & Kapodistrian University of Athens, PO 14561, Nikis 2, Athens, Greece

**Keywords:** bone defect, bone cement with screw augmentation, knee, arthritis, total knee arthroplasty, 3D-printing

## Abstract

The bone cement with screw augmentation (BCSA) technique is a valuable adjunct in complex total knee arthroplasty (TKA) cases with compromised bone stock. This surgical strategy enhances fixation in tibial and femoral defects, provides better load distribution, reduces micromotion, and improves the longevity of implants. The aim of this article is to explore the role of the BCSA technique, describe the technique itself, and review its outcomes specifically in the context of TKA. Additionally, we present a clinical case of an 80-year-old female patient with end-stage osteoarthritis and a severe tibial bone defect, who was treated with primary TKA using a 3D-printed cutting technique. The bone defect was managed using the BCSA technique with excellent imaging and clinical outcomes.

## Introduction

Total knee arthroplasty (TKA) is a common surgery to restore knee function and relieve pain [1]. Stable implant fixation is key to preventing loosening and ensuring long-term success. Good bone quality supports implant stability through osseointegration or secure cement bonding [2]. However, fixation can be difficult in osteoporotic bone or significant bone defects, where standard cemented techniques may fail [3]. Various methods manage bone loss in primary TKA: bone grafts (auto/allografts), cement (alone or with screws), metal augments, wedges, structural allografts, and porous materials like tantalum [4]. Using polymethylmethacrylate (PMMA) cement with screws improves fixation by enhancing cement interlock and providing mechanical support [4].

Bone defects in TKA fall into two main types: contained (central) defects in valgus knees, where the bone rim supports the implant, and uncontained (peripheral) defects in varus knees, where rim loss limits support [5]. The AORI classification is commonly used to assess these defects by size, severity, and location [5].

Careful pre-operative templating is crucial for managing bone defects and selecting the best method to balance flexion and extension gaps. Options include cement (with or without screws), bone grafting, or metal augments [6]. Defects ≤5 mm are usually treated with cement alone. For defects 5–10 mm (AORI type IIA), the BCSA technique using cancellous screws is effective. Defects >10 mm should not rely on cement and screws alone due to risk of weakness, failure, loosening, and thermal bone damage from excess cement [6, 7].

Follow-up radiographs may sometimes reveal early signs of implant loosening, indicated by the presence of radiolucent lines at the bone - cement interface. According to the literature, three studies [8–10] have analysed the progression of these radiolucent lines, which are frequently observed postoperatively and often precede implant loosening requiring revision surgery, as shown in Table 1.

**Table 1:** The table summarizes the risk of loosening after bone cement augmentation based on the size of the bone defect

**Study**	**Patient/case details**	**Bone defect size**	**Follow-up duration**	**Key findings**	**Outcomes**
Lotke *et al.* [8]	59 knees with bone defects	10–20 mm (*n* = 33) or > 20 mm (*n* = 23)	Mean 7.1 years	Non-progressive radiolucent lines at bone-cement interface in 43 knees.	Implant loosening in 1 patient, which was revised. No failure in the rest.
Dorr *et al.* [9]	54 patients with AORI Type 1 bone defects	AORI Type 1 defects	7 years	Cement used in Type 1 defects; good outcomes in 53 cases, failure in 1 case (loosening).	Good outcomes in most cases, failure in 1 due to loosening.
Ritter [10]	57 patients with tibial defects	9 mm mean defect	Minimum 3 years	Non-progressive radiolucency at the bone-cement interface in 25% of cases.	No failures observed.

## Case report/surgical technique

**Figure 1:**
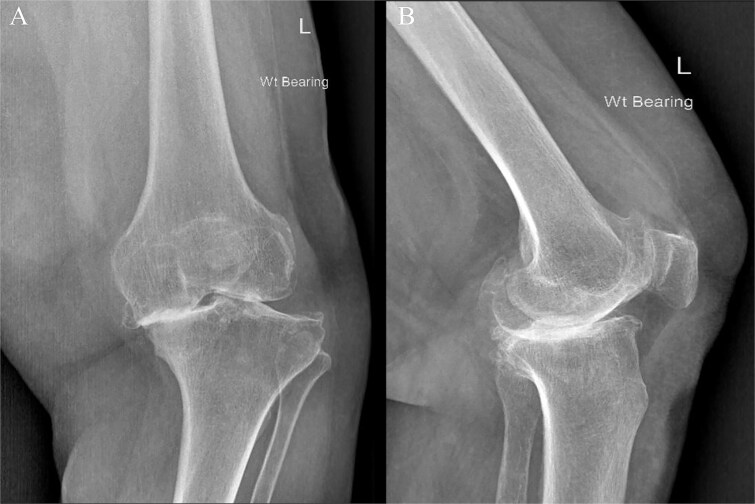
Preoperative face (A) and profile (B) X-rays showing end-stage knee arthritis with varus deformity and severe collision between the medial femoral and tibial condyles.

**Figure 2:**
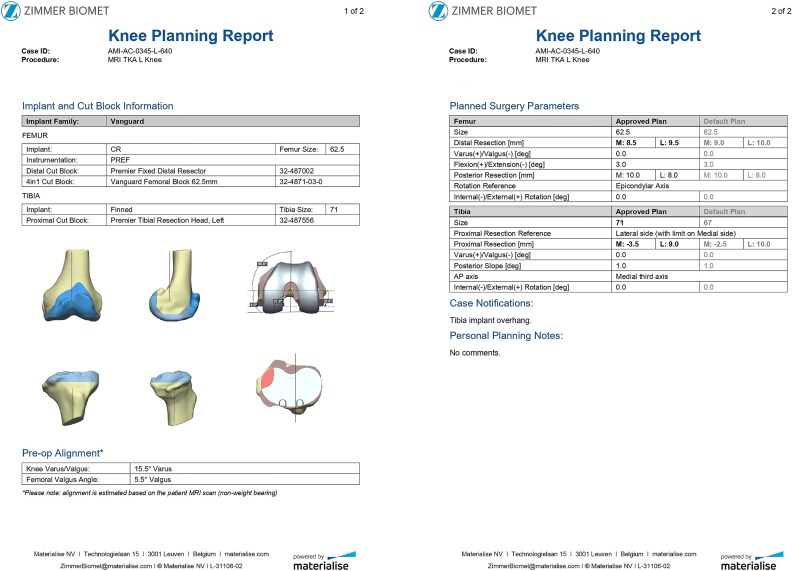
Preoperative plan presenting the medial tibial bone defect (orange color), the varus alignment of the native knee and the proposed femoral ant tibial cuts.

An 80-year-old woman presented with severe left knee pain, disability, and recurrent swelling. X-rays showed advanced osteoarthritis (Kellgren-Lawrence stage IV) with joint space narrowing, varus deformity, and a large medial tibial plateau defect (Fig. 1). The potential bone defect on the medial plateau, which could result in an unsupported region of the tibial component, was identified and highlighted in orange on the pre-operative templating plan. Additionally, a 15.5° varus deformity in the alignment of the native knee was observed (Fig. 2). Patient-specific, 3D-printed surgical guides (Zimmer Biomet), created from MRI-based models, were used for precise femoral and tibial resections.

The patient was positioned supine on the operating table, and a pneumatic tourniquet was applied and inflated at the proximal left thigh. A 12-cm midline skin incision was made, followed by a medial parapatellar arthrotomy. Precise femoral and tibial bone cuts were performed using patient-specific, 3D-printed cutting guides (Zimmer Biomet), following the pre-operative plan provided by the manufacturer.

**Figure 3:**
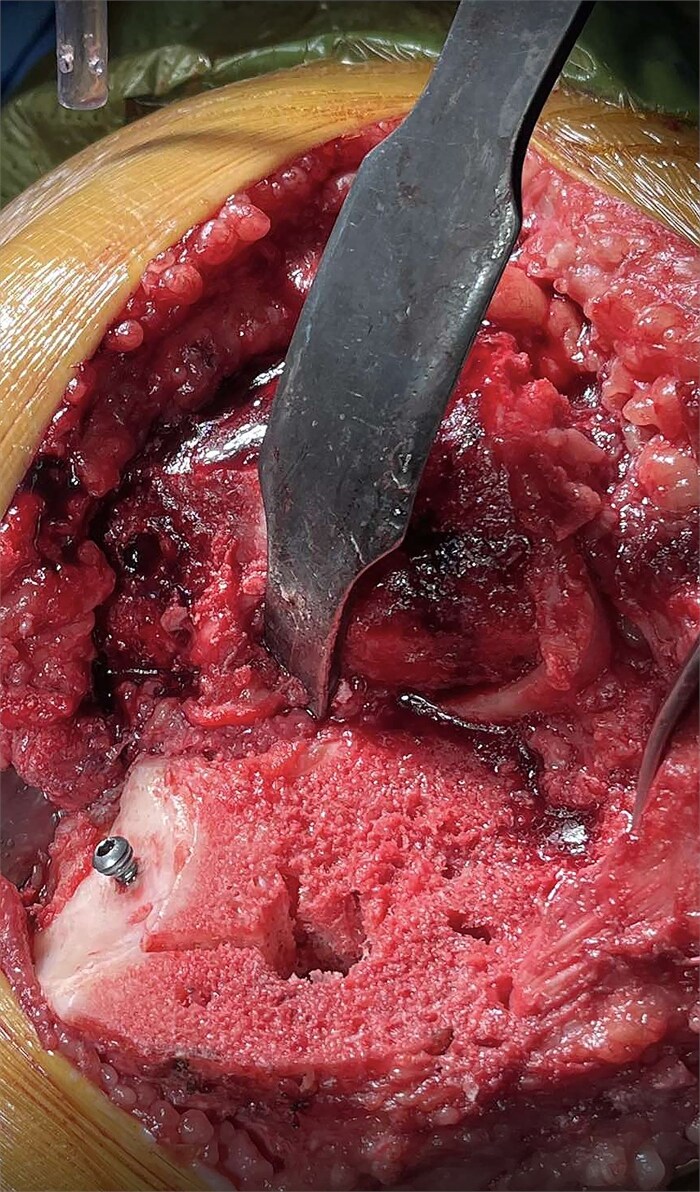
An intraoperative photograph showing the tibia after cuts made with customized 3D-printed guides. The non-contained 9.45 mm tibial bone defect was treated using the BCSA technique. Mechanical stability was ensured by the insertion of a 3.5 mm stainless steel cancellous screw.

Intraoperatively, around 9-10 mm was noted between the medial plateau rim and the trial tibial implant, classifying the defect as Anderson Orthopedic Research Institute (AORI) Classification type IIA. Consequently, the bone cement with screw augmentation (BCSA) technique [10] was selected to address the defect and reinforce the medial plateau. Multiple drill holes were created within the defect area using a 2.7 mm drill bit. A single 3.5 mm stainless steel cancellous screw was inserted to provide mechanical support (Fig. 3). PMMA bone cement was then injected into the drill holes and the entire defect area. Excess cement was removed after the tibial component was firmly seated in place.

The Vanguard knee system was implanted, comprising a 62.5 mm primary femoral component and a 71 mm primary tibial modular tray, both fixed with PMMA cement. A 12 mm ultra-high-molecular-weight polyethylene insert was positioned between the femoral and tibial components to complete the reconstruction.

## Discussion

Accurate preoperative classification of bone defects is often difficult, and their variability requires the use of multiple surgical strategies. Custom implants can address extensive bone loss but are associated with high financial costs. Conversely, bone cement remains a cost-effective option, though it is generally suitable only for managing small to moderate bone defects [11]. Bone grafts—whether autograft or allograft—carry inherent risks, including nonunion and disease transmission, limiting their desirability in TKA procedures [12]. Metal augments help fill bone voids but typically require additional cement for secure fixation.

Another option is using metaphyseal-filling devices, such as sleeves or tantalum cones, which are press-fit for bone ingrowth and structural support [13]. Metaphyseal sleeves can address bone loss in both the tibia and femur and lower the risks of collapse, nonunion, and disease transmission compared to allografts, though they do not promote bone regeneration [13]. In this case, a modular extension stem was added to the tibial baseplate to improve fixation and lower the risk of aseptic loosening [14].

The BCSA technique represents a practical and reliable method for addressing moderate bone defects. A 2021 cross-sectional study by Özcan Ö *et al.* reported excellent clinical outcomes in 37 knees of 28 patients treated with the BCSA technique for moderate tibial defects [4]. The mean Hospital for Special Surgery score at final follow-up (ranging from 28 to 75 months) was 88.0 ± 7.5, demonstrating the technique's effectiveness, even in obese patients with body mass index (BMI) > 30 [4]. Similarly, a 2023 case series by Alasaad H *et al*. assessed four patients with severe knee arthritis and significant medial tibial bone loss [15]. Their findings showed clear improvement in clinical and functional outcomes, based on the WOMAC score, during a 12- to 24-month follow-up period [15]. Earlier work by Ritter MA *et al.* also supports the efficacy of the BCSA technique [16]. In their study of 57 patients with tibial defects ranging from 4 to 13 mm, no failures were reported over a 13-year follow-up period [16]. Additionally, Lotke PA *et al.* reported only two failures in 59 patients treated with BCSA for tibial defects measuring 10 to 20 mm in depth [8].

**Figure 4:**
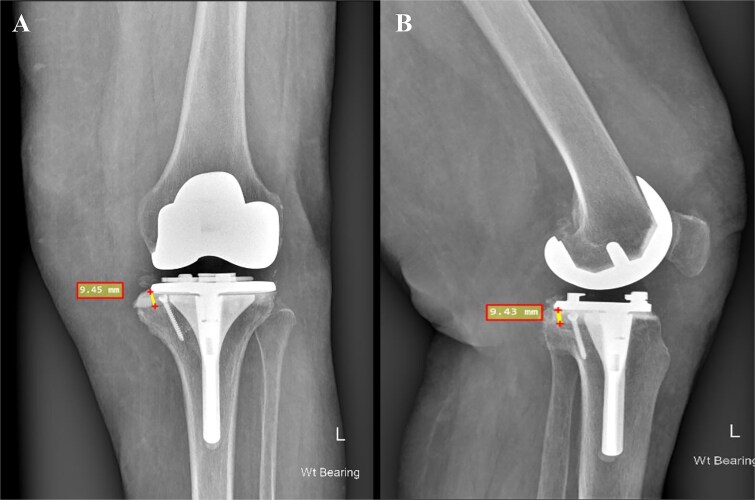
Postoperative face (A) and profile (B) X-rays depicting a medial tibial bone defect ~9.45 mm from the baseplate. The 3.5 mm cancellous screw is indicative of the applied BCSA technique.

In the present case, the BCSA technique was selected as the most suitable based on the pre-operative plan. The rationale for deeming BCSA the most appropriate option among others included the following: (i) the defect did not exceed 15%–20% of the tibial surface area, (ii) the technique permitted the use of standard primary TKA systems rather than necessitating revision prostheses as with metal augments, (iii) this method would preserve existing bone and soft tissue attachments, thereby facilitating both the planned primary and even a potential revision procedure, and (iv) the patient was obese with a BMI exceeding 30 and a diagnosis of established osteoporosis. Consequently, the use of excessive hardware combined with additional metal augments would elevate the mechanical load and increase the risk of future periprosthetic fracture. Our patient exhibited a bone defect of ~9.45 mm following the final tibial cuts (Fig. 3). Using cement alone in such a defect could have resulted in cement lamination, shrinkage, cracking, and early mechanical failure [17]. In our view, the application of the BCSA technique in this patient allowed for optimal cement pressurization within the defect, enhancing implant stability and fixation. At the 2-year follow-up, the patient showed excellent radiographic results (Fig. 4) and satisfactory clinical recovery (Video 1).

## Conclusion

Inadequate bone stock is a major challenge in primary TKA. The BCSA technique provides a safe, simple, and cost-effective solution. It is a reliable alternative for moderate bone defects, especially compared to costly options like tantalum implants, metal augments, or allografts. BCSA also preserves more bone, aiding potential future revisions.

## Supplementary Material

Video_rjaf884
